# Exploring Early Neurodegeneration Through Fasting-Induced Metabolic Signatures and High-Sensitivity Biomarkers

**DOI:** 10.3390/cimb48040358

**Published:** 2026-03-28

**Authors:** Francesco Cacciabaudo, Luisa Agnello, Caterina Maria Gambino, Giulia Accardi, Anna Masucci, Martina Tamburello, Roberta Vassallo, Marcello Ciaccio

**Affiliations:** 1 Institute of Clinical Biochemistry, Clinical Molecular Medicine, and Clinical Laboratory Medicine, Department of Biomedicine, Neurosciences and Advanced Diagnostics, University of Palermo, 90127 Palermo, Italy; francesco.cacciabaudo@unipa.it (F.C.); luisa.agnello@unipa.it (L.A.); caterinamaria.gambino@unipa.it (C.M.G.); anna.masucci@unipa.it (A.M.); martina.tamburello@unipa.it (M.T.); roberta.vassallo03@unipa.it (R.V.); 2 Department of Laboratory Medicine, University Hospital Paolo Giaccone, 90127 Palermo, Italy; 3 Laboratory of Immunopathology and Immunosenescence, Department of Biomedicine, Neurosciences and Advanced Diagnostics, University of Palermo, 90127 Palermo, Italy; giulia.accardi@unipa.it

**Keywords:** intermittent fasting, neurodegenerative diseases, neurometabolic pathways, mitochondrial quality control, neuroinflammation, ultrasensitive biomarkers, precision nutrition

## Abstract

Neurodegenerative diseases (NDs) are increasingly considered neurometabolic disorders driven by early mitochondrial dysfunction, neuroinflammation, and synaptic alterations that precede clinical symptoms. This review summarises pre-clinical and experimental evidence suggesting that intermittent fasting (IF) may influence these early pathogenic processes by promoting metabolic switching, enhancing autophagy and mitochondrial quality control, and modulating neuroimmune pathways. We discuss recent advances in biomarker research supporting the early detection of neurodegenerative changes, including ultrasensitive analytical platforms that can identify neuronal, glial, and synaptic injury during preclinical stages. By integrating these biomarker developments with findings from human and experimental intermittent fasting studies, we highlight how high-sensitivity assays provide quantifiable insights into the neurometabolic effects of fasting. Furthermore, we discuss how precision nutrition strategies incorporating multimarker panels, phenotypic and epigenetic signatures, and longitudinal multi-omics profiling may facilitate personalised intermittent fasting protocols and improve monitoring of biological responses. Overall, these findings underscore the relevance of a clinical biochemistry perspective integrating advanced biomarker technologies to evaluate the neurometabolic effects of intermittent fasting as a potential early neuroprotective strategy for individuals at risk of neurodegeneration.

## 1. Introduction

**Figure 1:**
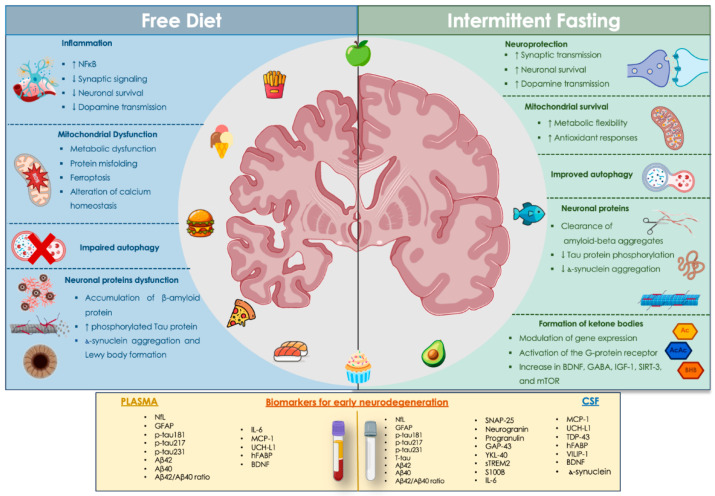
Schematic overview of early neurodegenerative mechanisms under a free diet and the neuroprotective metabolic and synaptic adaptations promoted by IF, with relevant circulating biomarkers. The left panel depicts biological processes associated with a free diet, including increased inflammatory signalling, mitochondrial dysfunction, impaired autophagy, and the accumulation of misfolded neuronal proteins. The right panel summarises the metabolic and neuroprotective adaptations linked to IF, such as enhanced mitochondrial resilience, improved autophagy, modulation of gene expression, and increased neurotrophic signalling, mainly supported by experimental and preclinical evidence. The lower section displays representative circulating biomarkers measurable in plasma and CSF that indicate neuronal injury, synaptic dysfunction, inflammation, and protein aggregation during early neurodegeneration. These biomarkers serve as measurable indicators of the underlying pathological processes rather than direct mechanistic causes. Abbreviations: NF-κB (Nuclear Factor kappa-light-chain-enhancer of activated B cells); CSF (cerebrospinal fluid); BHB (β-hydroxybutyrate); BDNF (Brain-Derived Neurotrophic Factor); GABA (Gamma-aminobutyric Acid); IGF-1 (Insulin-like Growth Factor 1); SIRT-3 (Sirtuin 3); mTOR (Mechanistic Target of Rapamycin); NfL (Neurofilament Light Chain); GFAP (Glial Fibrillary Acidic Protein); p-Tau181, p-Tau217, p-Tau231 (Phosphorylated Tau protein); t-Tau (Total tau); Aβ42/Aβ40 (Amyloid-beta 42/Amyloid-beta 40); IL-6 (Interleukin-6); MCP-1 (Monocyte Chemoattractant Protein-1); UCH-L1 (Ubiquitin C-terminal Hydrolase L1); hFABP (Heart-type Fatty Acid-Binding Protein); SNAP-25 (Synaptosomal-associated Protein 25); GAP-43 (Growth-associated Protein 43); YKL-40 (Chitinase-3-like protein 1); sTREM2 (Soluble Triggering Receptor Expressed on Myeloid cells 2; S100B (S100 calcium-binding protein B); TDP-43 (TAR DNA-binding protein 43); VILIP-1 (Visinin-like Protein 1). Created in BioRender. Ciaccio, M. (2026) https://BioRender.com/au5k56i (accessed on 22 March 2026).

Neurodegenerative diseases (NDs), including Alzheimer’s disease (AD), other dementias, and Parkinson’s disease (PD), are a rapidly expanding global health challenge, primarily driven by population ageing and demographic shifts. These conditions are major contributors to disability and mortality worldwide, with AD and other dementias alone accounting for over 43 million cases globally as of 2016, and prevalence continues to rise [1]. The Global Burden of Disease Study 2021 found that disorders of the nervous system, including NDs, are now the leading cause of disability-adjusted life years (DALYs) worldwide, accounting for more than 40% of DALYs and over 443 million DALYs in 2021 [2]. Recent evidence increasingly supports the idea that NDs originate from early, interconnected disruptions in metabolic homeostasis, mitochondrial resilience, neuroinflammation, and synaptic integrity [3,4]. A significant amount of research shows that metabolic changes, including dysregulated calcium levels, compromised mitochondrial function, and defective autophagy, occur during the asymptomatic stages of AD and mild cognitive impairment (MCI) [5]. Microglial studies further suggest that mitochondrial dysfunction occurs before overt neuroinflammation and is associated with a shift from oxidative phosphorylation to glycolysis [6]. Early cognitive vulnerability is also influenced by ethnicity, sex, lifestyle, and comorbidities, highlighting the multifactorial nature of preclinical neurodegeneration [7]. Parallel findings in PD highlight the early involvement of lipid dysregulation in mitochondrial α-sinuclein (α-syn) interactions and ferroptosis [8]. At the same time, shared molecular pathways, such as oxidative stress, neuroinflammation, and protein misfolding, are increasingly recognised across synucleinopathies and amyloid-related disorders [9]. Collectively, this evidence suggests that mitochondrial dysfunction is a hallmark of early neurodegenerative processes, and even small impairments have measurable effects on neuronal health [10]. Within this rapidly evolving landscape of biology and analysis, intermittent fasting (IF) has emerged as a promising metabolic intervention that modulates pathways implicated in early neurodegeneration. Preclinical and clinical studies show that IF promotes metabolic switching toward ketone use, boosts autophagy, improves mitochondrial function, and influences neuroinflammatory and synaptic pathways via mediators such as β-hydroxybutyrate (BHB), short-chain fatty acids (SCFAs), and Brain-Derived Neurotrophic Factor (BDNF) [11,12]. Clinically, IF has been linked to improvements in body weight, lipid profiles, insulin sensitivity, and cardiovascular health across various populations. Emerging conceptual models in precision nutrition suggest that individual metabolic profiles, visceral fat distribution, sex differences, and epigenetic markers may help tailor personalised IF protocols [13]. Progress in clinical biochemistry has significantly enhanced the ability to identify these early alterations using plasma biomarkers. Neurofilament light chain (NfL), Glial Fibrillary Acidic Protein (GFAP), phosphorylated Tau (p-Tau), Synaptosomal-associated protein 25 (SNAP-25), and neurogranin now allow sensitive assessment of axonal injury, astroglial activation, and synaptic dysfunction years before symptoms emerge [14,15,16]. Their diagnostic and prognostic value has been consistently demonstrated across preclinical and prodromal stages of AD [17,18]. Large multicentre studies further confirm that multi-marker panels outperform single analytes by providing a more comprehensive view of amyloid, Tau, axonal, and astroglial activity [19]. These advances have been driven by ultrasensitive analytical platforms capable of multiplexed, low-volume detection with high analytical sensitivity, features essential for identifying subtle biological changes during early disease stages [20,21]. This review combines three rapidly evolving fields: early mechanisms of neurodegeneration, ultrasensitive biomarker technologies, and the molecular and clinical impacts of IF to provide an integrated view relevant to clinical biochemistry. Connecting mechanistic evidence with advances in diagnostics and personalised metabolic strategies provides a translational conceptual basis for assessing IF as an early neuroprotective intervention in preclinical neurodegeneration. Notably, evaluating circulating biomarkers directly from plasma offers a practical, clinically significant approach, enabling minimally invasive monitoring of IF-related biological effects in individuals at the earliest signs of cognitive decline. Figure 1 provides a visual summary of the interconnected metabolic, mitochondrial, inflammatory, and synaptic changes underlying early neurodegeneration. It also emphasises how IF could help mitigate these processes via neuroprotective and metabolic pathways.

In this context, the term “fasting-induced metabolic signatures” refers to coordinated and interconnected changes across key metabolic pathways and circulating mediators, such as increased ketone bodies (BHB), reduced insulin/IGF-1 signalling, modulation of mTOR and SIRT3 activity, and attenuation of inflammatory pathways, rather than comprehensive metabolomics-defined profiles.

## 2. Metabolic Foundations of Intermittent Fasting

IF has become a metabolic approach that induces notable physiological and neurological changes through various fasting protocols, such as Alternate-day Fasting (ADF), Time-restricted Feeding (TRF), and other structured methods. In different populations, especially those with obesity or metabolic syndrome, these fasting regimens consistently improve metabolic health by reducing body fat, improving lipid profiles, and lowering blood pressure [22].

IF encompasses a heterogeneous group of dietary strategies that primarily modulate food intake over time rather than through continuous caloric restriction. These approaches can be broadly classified into three main paradigms: ADF, which alternates prolonged fasting periods (typically 24–36 h) with days of ad libitum feeding; periodic fasting, exemplified by the 5:2 regimen, in which severe energy restriction (approximately 500–600 kcal) is applied on two non-consecutive days per week; and time-restricted eating, which limits daily food intake to a defined time window, most commonly 8 h, followed by a fasting period of approximately 16 h [23,24]. Fasting phases may involve complete caloric abstinence, allowing only non-caloric beverages such as water, unsweetened tea, or black coffee, or modified protocols that permit a reduced energy intake, generally 20–40% of habitual caloric intake. Conversely, feeding periods are often implemented on an ad libitum basis without mandatory calorie counting, although in controlled research settings, both energy intake and macronutrient composition are frequently standardised [25]. Notably, the operational definition of IF varies substantially across studies. In the literature, the term IF is often used as an umbrella concept encompassing several temporal dietary strategies, including TRF and intermittent energy restriction. Intermittent energy restriction is commonly defined as consuming ≤800 kcal on one to six days per week, with intervention durations ranging from short-term protocols of three weeks to long-term interventions lasting up to twelve months. Moreover, implementation strategies vary widely, from self-directed adherence to highly supervised programmes supported by registered dietitians. This marked heterogeneity, particularly regarding the degree of caloric restriction during fasting periods and the level of control during feeding phases, poses significant challenges for cross-study comparisons and for translating IF protocols into routine clinical practice [26]. The central mechanism behind these benefits is the metabolic shift from glucose utilisation to ketone body production during fasting. As hepatic fatty acid oxidation increases, circulating ketone bodies, primarily BHB, rise to serve as alternative energy sources [27,28]. Beyond their role in energy, ketone bodies act as powerful signalling molecules. BHB influences gene expression through post-translational modifications, activates G protein-coupled receptors, and affects cellular processes independently of its metabolic role [29,30]. The cyclical pattern of fasting and feeding triggers evolutionarily conserved stress–response pathways during fasting, followed by mechanisms that promote growth and plasticity during refeeding. Major molecular mediators of these cycles include ketone bodies, BDNF, γ-aminobutyric acid (GABA), Growth hormone (GH), insulin-like growth factor-1 (IGF-1), Sirtuin 3 (SIRT3), and mTOR, which collectively enhance stress resilience, synaptic plasticity, neurogenesis, and mitochondrial function [31,32]. These coordinated responses enhance insulin sensitivity, regulate IGF-1 signalling, support mitochondrial bioenergetics, and contribute to overall metabolic balance [33,34,35]. Taken together, these metabolic mechanisms indicate that IF represents a systemic metabolic intervention rather than a purely nutritional strategy, with potential implications for early neuroprotection. The signalling pathways activated by IF converge with multiple biological processes involved in the initiation and progression of neurodegenerative pathology [36,37].

## 3. Metabolic Signatures of Fasting as Modulators of Early Neurodegenerative Biomarkers

**Figure 2:**
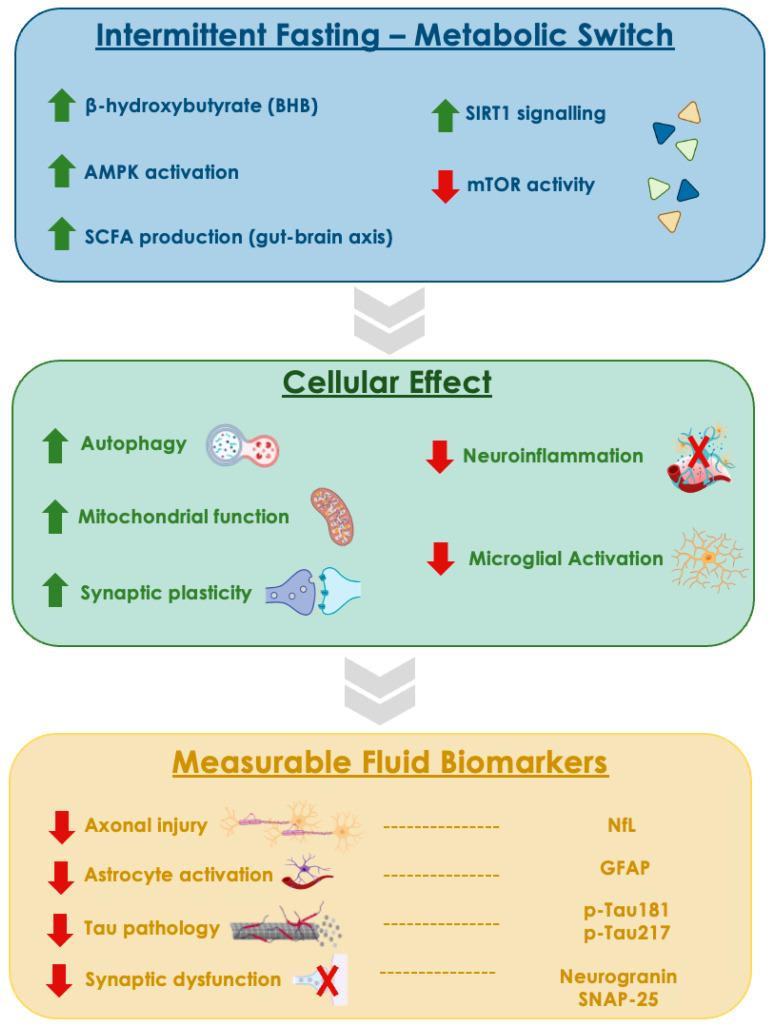
Conceptual framework linking intermittent fasting-induced metabolic mechanisms to cellular pathways and measurable fluid biomarkers of early neurodegeneration. Abbreviations: BHB, β-hydroxybutyrate; AMPK, AMP-activated protein kinase; SIRT1, sirtuin-1; mTOR, mechanistic target of rapamycin; SCFA, short-chain fatty acids; NfL, neurofilament light chain; GFAP, glial fibrillary acidic protein; p-Tau181, phosphorylated tau at threonine 181; p-Tau217, phosphorylated tau at threonine 217. Created in BioRender. Ciaccio, M. (2026) https://BioRender.com/eleqd1x (accessed on 22 March 2026).

Emerging evidence supports a pathophysiological continuum in which IF induces a metabolic switch, characterised by BHB production, AMP-activated protein kinase (AMPK) activation, mTOR inhibition, modulation of SIRT1 signalling, and altered SCFA profiles, that converges on molecular pathways implicated in early neurodegeneration [38,39,40]. In preclinical models of AD and PD, these mediators have been associated with reduced amyloid-β accumulation, attenuated tau phosphorylation, reduced microglial activation and pro-inflammatory cytokine release, enhanced autophagic clearance of protein aggregates, and improved synaptic plasticity, partly mediated by BDNF upregulation. Importantly, these pathways may exert context-dependent effects across distinct neural cell populations, including neurons, astrocytes, and microglia, which display different metabolic and inflammatory responses to energetic stress. The gut–brain axis appears to represent a complementary pathway, as IF-induced increases in SCFAs, particularly butyrate, may cross the blood–brain barrier and influence glial and inflammatory homeostasis [41]. However, translating these mechanistic effects into measurable changes in human fluid biomarkers remains limited. The most consistent clinical signal comes from multiple sclerosis, where a 6-month adapted ketogenic diet significantly reduced serum NfL, suggesting reduced axonal injury [42,43]. Intermittent calorie restriction has also been linked to reversing accelerated metabolic ageing, while continuous calorie restriction did not produce similar results, suggesting that metabolic cycling, rather than caloric deficit alone, may be important [44,45]. Importantly, no controlled human study to date has demonstrated that IF directly alters plasma Amyloid-β 42/Amyloid-β 40 (Aβ42/40) ratios, phosphorylated tau species (p-Tau181 or p-Tau217), or GFAP, as predefined intervention endpoints [46,47,48]. Moreover, an acute fasting state may transiently influence circulating biomarker concentrations, introducing potential confounding in cross-sectional designs. Available data also suggest a duration-dependent effect, as short-term fasting interventions (e.g., 7 days) have failed to alter NfL levels, implying that sustained or cyclic exposure may be required to engage biomarker-relevant neurobiological pathways [49]. The conceptual links between fasting-induced metabolic mediators, cellular pathways, and measurable biomarkers of neurodegeneration are summarised schematically in Figure 2. Taken together, current evidence supports a biologically plausible link between IF-induced metabolic mediators and neurodegeneration-related pathways, but robust longitudinal human data directly linking fasting signatures to established plasma or cerebrospinal fluid (CSF) biomarkers of AD remain insufficient.

## 4. Metabolic and Neuroinflammatory Pathways in Early Neurodegeneration

Fasting protocols, such as ADF and TRF, are important for neurodegenerative diseases. These mechanisms are significant because histone deacetylase inhibition and improved mitochondrial efficiency, mediated by ketone body signalling and SIRT3 activation, have been linked in preclinical models to reduced tau phosphorylation and enhanced synaptic resilience [50,51]. However, direct human evidence linking circulating BHB concentrations to long-term changes in plasma or CSF biomarkers remains limited [52]. Notably, several of these pathways converge with early pathophysiological aspects of neurodegeneration. In particular, mTOR inhibition and SIRT3 activation promote autophagic clearance of misfolded proteins, potentially affecting amyloid-β and tau turnover; BDNF signalling supports synaptic integrity, possibly modulating synaptic biomarkers such as neurogranin; and attenuation of neuroinflammatory pathways may reduce astroglial activation, as reflected in GFAP dynamics [53,54,55,56]. Alongside these intracellular metabolic adaptations, increasing evidence suggests that mitochondrial dysfunction and metabolic stress alter neuroimmune signalling and glial function during early stages of disease. A growing body of research has enhanced our understanding of how metabolic changes, mitochondrial damage, and neuroinflammation collectively influence the initial phases of neurodegeneration. In AD and MCI, metabolic dysfunction appears to be a key early factor, with changes in calcium balance, mitochondrial failure, and impaired autophagy contributing to pathology during the asymptomatic preclinical stage [57]. Microglial biology offers a clear example of this connection: mitochondrial dysfunction has been shown to precede neuroinflammation, initiating a metabolic shift from oxidative phosphorylation to glycolysis during proinflammatory activation. Parallel evidence from PD highlights the role of abnormal lipid metabolism and the dynamic crosstalk between mitochondria and α-syn [58]. Dysregulated lipids increase α-syn aggregation, disrupt mitochondrial and endoplasmic reticulum function, and promote ferroptosis, linking metabolic imbalance to neuronal vulnerability. Importantly, shared molecular pathways, including oxidative stress, neuroinflammation, protein misfolding, and mitochondrial impairment, are now recognised across synucleinopathies and amyloidogenic disorders, despite their distinct pathological features [59]. Similar principles apply to early neuroinflammatory conditions, such as multiple sclerosis, in which metabolic dysregulation influences microglial phenotypes and impacts tissue repair via pathways related to iron metabolism, fatty acids, and amino acid availability. Across these disorders, mitochondrial dysfunction is a common hallmark. Because neurons have extremely high energy requirements, even minor defects in mitochondrial respiration can lead to oxidative stress, energy shortages, and abnormal protein processing [50]. These findings have shaped strategies to boost mitochondrial resilience using antioxidants, modulators of mitochondrial dynamics, and gene-based therapies. Efforts to find reliable mitochondrial biomarkers are also progressing, aiming to detect pathological changes before clinical symptoms appear [45]. Taken together, these findings support a model in which metabolic stress, mitochondrial dysfunction, and neuroimmune signalling act as interconnected drivers of early neurodegenerative processes, providing mechanistic links between metabolic interventions such as IF and pathways relevant to biomarker-detectable disease progression.

## 5. Plasma and CSF Biomarkers for Early Neurodegeneration

The rapid growth of biomarker research is changing strategies for the early detection of NDs, fuelled by advances in multi-domain characterisation and a growing focus on clinical applications. Among the most well-established biomarkers, NfL has become a reliable marker of axonal injury, with strong reproducibility across platforms. It has been demonstrated that CSF Total Tau (t-Tau), p-Tau, Amyloid-β 42 (Aβ42), NfL, and plasma t-Tau closely correlate with AD pathology, and that higher levels of plasma and CSF NfL are linked to reduced ^18^F-fluorodeoxyglucose uptake in brain regions vulnerable to early degeneration, especially in Aβ–positive individuals [60]. GFAP has also gained prominence as a sensitive biomarker of astrocytic activation. Studies report strong CSF–plasma correlations, and trajectory modelling shows that plasma GFAP, Aβ42/Aβ40, and p-Tau231 exhibit the earliest rates of change at CSF-defined thresholds of amyloidosis and tauopathy, supporting their relevance for preclinical disease monitoring [61,62,63]. SNAP-25, neurogranin, and progranulin are increasingly being incorporated into the evolving Amyloid/tau/synaptic dysfunction/neurodegeneration framework biomarker classification, alongside promising candidates such as Chitinase-3-like protein 1 (YKL-40), Growth-Associated Protein 43 (GAP-43), Visinin-like protein 1 (VILIP-1), Triggering Receptor Expressed on Myeloid Cells 2 (TREM2), IGF-1, Heart-type Fatty Acid-Binding Protein (hFABP), Monocyte Chemoattractant Protein-1 (MCP-1), TAR DNA-binding Protein 43 (TDP-43), and BDNF [46]. SNAP-25 and neurogranin offer high predictive value for synaptic impairment, and combined CSF measurements of SNAP-25 and p-tau231 provide strong discrimination between healthy controls and individuals with preclinical AD [64]. Multi-analyte panels including GFAP, YKL-40, sTREM2, S100 calcium-binding protein B (S100B), and Interleukin-6 (IL-6) have shown associations with neurodegeneration biomarkers in cognitively normal individuals, suggesting that glial activation happens before measurable cognitive decline [65]. Recent studies highlight that YKL-40 and TREM2 remain promising candidates for future validation, although further research is needed before they can be used routinely in diagnostics [66]. Table 1 provides an overview of the major fluid biomarkers discussed here, summarising their biological roles and their diagnostic and prognostic relevance in early neurodegeneration, together with supporting evidence from recent literature. Notably, biomarkers reflecting metabolic and oxidative stress remain relatively underdeveloped, highlighting an important opportunity for future research to expand the biological scope of current diagnostic models.

A major conceptual breakthrough has emerged from multi-biomarker approaches. Large international studies show that plasma panels combining Aβ42/Aβ40, p-Tau181, GFAP, and NfL outperform single-analyte methods, effectively differentiating AD from controls and non-AD dementias [67]. The field is increasingly recognising that combining multiple biomarkers offers a more complete picture of amyloid and tau pathology, axonal injury, and astrocytic activation, boosting diagnostic confidence and staging accuracy. Technological advances have further sped up these developments. Improvements in ultrasensitive analytical platforms now allow high-performance detection of biomarkers in plasma, serum, and neuron-derived exosomes, supporting minimally invasive, scalable methods suitable for long-term monitoring [68]. Together, these advances in biomarker discovery, analytical technologies, and clinical validation are driving a paradigm shift toward detecting neurodegenerative changes years or even decades before symptoms appear. This change enables earlier therapeutic intervention and supports a shift from reactive symptom management to proactive disease modification and precision medicine.

The molecular pathways described above suggest a mechanistic link between energy-sensing networks and fluid biomarkers of early neurodegeneration. Activation of AMPK and inhibition of mTOR promote autophagic flux and proteostasis, mechanisms directly relevant to amyloid-β clearance and tau phosphorylation dynamics, thereby intersecting with Aβ42/Aβ40 ratios and p-Tau181/p-Tau217 trajectories. SIRT1 and Nuclear factor erythroid 2–related factor 2 activation modulate oxidative stress responses and mitochondrial resilience, potentially influencing axonal integrity, as reflected by NfL [69]. Enhanced mitophagy and mitochondrial quality control may indirectly affect neuronal injury markers by limiting oxidative damage and ferroptotic signalling. At the synaptic level, restoration of proteostasis and improved mitochondrial function may theoretically modulate synaptic biomarkers such as SNAP-25 and neurogranin, while metabolic and inflammatory signalling pathways converge on astrocytic and microglial activation states, intersecting with GFAP, TREM2, and YKL-40 dynamics [70,71]. In this context, fasting-induced mediators such as BHB, AMPK activation, mTOR suppression, SIRT1 signalling, SCFA production, and downstream modulation of the PI3K/Akt and MAPK pathways form a biologically coherent axis linking systemic metabolic switching to biomarker-relevant neurobiological processes. However, while these intersections are mechanistically plausible, direct human evidence of consistent biomarker modulation along this axis remains limited, underscoring the need for longitudinal translational studies that integrate metabolic mediators and validated Amyloid-Tau-Neurodegeneration framework (AT(N)) biomarkers [72].

## 6. Do Fasting-Induced Metabolic Mediators Measurably Modify Plasma/CSF Biomarkers?

The integration of IF into biomarker-driven models of early neurodegeneration requires a critical appraisal of the evidence linking fasting-induced metabolic mediators to measurable modulation of established fluid biomarkers. In humans, caloric restriction and IF have been consistently associated with improved insulin sensitivity, reductions in systemic inflammatory markers (e.g., C-reactive protein, IL-6), modulation of oxidative stress parameters, and favourable cardiometabolic changes. Limited interventional studies in neurological populations, particularly in multiple sclerosis, suggest potential reductions in inflammatory mediators and, in exploratory settings, in NfL [73]. However, robust longitudinal randomised trials demonstrating significant and sustained modulation of core AD plasma biomarkers, such as Aβ42/Aβ40 ratio, p-Tau181, p-Tau217, GFAP, or NfL, are currently lacking. By contrast, preclinical models provide mechanistic plausibility. In rodent models of AD, fasting or ketogenic interventions increase BHB levels, activate AMPK, inhibit mTOR signalling, enhance autophagic flux, and reduce amyloid-β deposition and tau hyperphosphorylation [74]. These pathways intersect with molecular mechanisms implicated in synaptic integrity (e.g., SNAP-25, neurogranin), astrocytic activation (GFAP), and microglial signalling (TREM2). Additionally, SCFAs, particularly butyrate, may cross the blood–brain barrier and exert epigenetic and anti-inflammatory effects that could theoretically influence trajectories of glial and inflammatory biomarkers [75]. Taken together, while caloric restriction and IF improve systemic metabolic and inflammatory profiles in humans, direct evidence for sustained modulation of validated plasma or CSF AT(N) biomarkers specifically attributable to fasting-induced metabolic switching, remains limited. Therefore, the proposed link between IF-induced ketone signalling and disease-modifying effects on established neurodegeneration biomarkers should be regarded as biologically coherent and mechanistically supported, yet not conclusively established at the clinical biomarker level [76]. Future longitudinal studies integrating metabolic mediators, BHB, SCFAs, and insulin signalling indices with fluid biomarker trajectories in well-characterised at-risk cohorts are needed to determine whether IF exerts true biomarker-modifying effects or primarily acts as a systemic metabolic regulator with indirect neurobiological implications.

## 7. Metabolic, Molecular, and Clinical Effects of IF

Growing evidence positions IF as a promising preventive and early therapeutic strategy that can reshape metabolic homeostasis and modulate molecular pathways relevant to cardiometabolic health and neurodegeneration. A recent systematic review and meta-analysis of 15 randomised controlled trials (RCTs) (n = 758) reported consistent improvements in body weight, total cholesterol, LDL cholesterol, and diastolic blood pressure [11]. It also highlighted a transient increase in triglycerides during short-term interventions (≤12 weeks) that normalised or reversed with longer protocols (>12 weeks), reflecting progressive metabolic adaptation. At the molecular level, IF activates AMPK, suppresses mTOR signalling, and enhances autophagic activity, leading to a metabolic shift toward fatty acid and ketone utilisation, modulation of the GH/IGF-1 axis, and regulation of the MAPK, Notch, and NF-κB pathways [34]. Neuroprotective effects are further supported by gut–brain interactions mediated by SCFAs, BHB, and BDNF, promoting neuronal resilience, synaptic plasticity, and mitochondrial function. Despite these encouraging findings, clinical evidence remains heterogeneous, with many studies limited by short follow-up, small sample sizes, non-standardised fasting regimens, and variable adherence, including non-negligible dropout rates in longer or more restrictive interventions, underscoring the need for more rigorous and clinically feasible trial designs. Long-term, well-controlled RCTs are essential to establish the efficacy of IF in the prevention and management of age-related diseases. Emerging research priorities include investigating sex-specific responses, given documented differences in body composition, insulin sensitivity, and lipid metabolism linked to age and hormonal status, as well as identifying validated biomarkers that reliably capture IF-induced autophagic and metabolic responses in humans. These differences may be influenced by factors including age, baseline body composition, adipose tissue distribution, and hormonal regulation. Accordingly, the existing literature on IF clinical trials highlights the importance of considering both protocol variability and sex-specific metabolic differences when evaluating the clinical applicability of fasting interventions. Further research is therefore required to determine the most effective fasting schedules and durations for different populations. Tailoring IF strategies to individual characteristics may ultimately enhance its effectiveness as a lifestyle intervention with potential cardiometabolic and neuroprotective benefits [13,35]. Personalised approaches are increasingly recognised, and stratification based on visceral fat ratio (VFR) suggests that individuals with high VFR may derive greater benefit from early time-restricted eating combined with structured exercise, whereas those with moderate VFR may require closer cardiometabolic monitoring when following alternate-day fasting protocols. Epigenetic variability is also emerging as a key determinant of individual responsiveness, involving DNA methylation patterns, histone modifications, and non-coding RNAs [36]. Ongoing clinical trials represent the next phase of IF research, moving toward mechanistic depth and metabolic precision. Notably, the IF and Stability Trial is evaluating triglyceride metabolic reprogramming in adults at high risk for type 2 diabetes, including individuals with prior gestational diabetes or a first-degree family history, using longitudinal metabolomics and hyperinsulinemic-euglycemic clamps. However, methodological heterogeneity, particularly regarding feeding–fasting schedules, intervention duration, and dietary context, remains a persistent limitation, reinforcing calls for standardised protocols to improve cross-study comparability. Collectively, these findings underscore the central role of intermittent metabolic switching from glucose to fatty acid and ketone oxidation as an evolutionarily conserved adaptive response. In this broader context, IF is increasingly proposed not as an isolated dietary intervention but as part of a structured Mediterranean dietary pattern that supports anti-inflammatory balance, provides high-quality nutrients, and stabilises cardiometabolic parameters, thereby enhancing fasting-related biochemical adaptations. Current priorities include developing digital adherence tools, biomarker-guided feeding windows, circadian-aligned fasting schedules, and long-term safety monitoring, all of which are essential for establishing standardised, Mediterranean-based IF protocols suitable for precision nutrition and the prevention of metabolic disease [77]. An overview of the main metabolic, molecular, and clinical effects of IF, as reported in key experimental and clinical studies, is summarised in Table 2.

## 8. Future Directions in IF: Biomarkers and Precision Neuro-Nutrition

Longitudinal, biomarker-driven studies are becoming essential for advancing our understanding of IF as a potential neuroprotective strategy. Evidence from preclinical and early human research shows that IF can enhance cognitive performance and reduce AD-related pathology, partly through metabolic and gut–brain interactions [78]. However, current clinical evidence remains limited and diverse, with most studies having small sample sizes, short follow-up durations, and varying fasting protocols. However, another study points out that future nutritional research on cognitive ageing must include long-term, repeated measurements to capture the dynamic changes in fasting-induced adaptations [79]. Extensive longitudinal profiling over multiple years has proven valuable in identifying actionable molecular pathways involved in metabolic and neurological physiology [80]. More efforts are being focused on customising metabolic interventions based on individual risk profiles, including genetically vulnerable groups such as Apolipoprotein E ε4 carriers, for whom targeted metabolic strategies show theoretical promise but still need validation through long-term human studies [81]. Emerging concepts like “smart neuro-nutrition”, which combine artificial intelligence (AI) with personalised dietary profiling, demonstrate how digital and computational tools could improve cognitive resilience through tailored metabolic modulation [82,83]. Currently, however, AI-guided precision nutrition in the context of NDs remains at an early and largely exploratory stage, and empirical evidence demonstrating clinical effectiveness is still limited. These developments depend heavily on advances in high-throughput multi-omics technologies and integrative data-science frameworks. Multi-omics profiling can reveal interindividual differences in dietary responses. At the same time, digital phenotyping and wearable devices are being explored as tools to monitor metabolic rhythms, feeding–fasting cycles, sleep patterns, and physiological markers in real-world settings [84,85,86]. However, most of these technologies are currently used in exploratory or feasibility studies rather than in validated clinical applications. These insights collectively suggest a future where IF is integrated into a broader precision neuro-nutrition framework. However, this vision should currently be regarded as a research avenue rather than an established clinical approach. Future studies combining longitudinal biomarker monitoring, multi-omics profiling, and digital health tools will be necessary to determine whether such methods can effectively influence early neurodegenerative processes.

## 9. IF Clinical Considerations: Safety and Contraindications

Although IF has gained attention as a potential metabolic strategy for promoting brain health, its implementation in older adults or individuals at risk of neurodegenerative disorders requires careful clinical assessment [87,88]. Older populations often present comorbidities, polypharmacy, and altered metabolic resilience, which may affect the safety and tolerability of fasting interventions. Potential risks include hypoglycemia in individuals with diabetes treated with insulin or insulin secretagogues, worsening of frailty or sarcopenia during prolonged caloric restriction, and possible interactions with medications that require food for optimal absorption or tolerability [89]. Furthermore, prolonged fasting protocols may not be suitable for individuals with low body mass index, advanced chronic illness, or a history of eating disorders. For these reasons, most clinical studies highlight the importance of medical supervision, gradual implementation of the protocol, and personalised assessment of nutritional status and metabolic risk before starting IF interventions. Future trials should include systematic safety monitoring, such as glycaemic control, body composition, and functional status, especially in older or vulnerable groups [90].

## 10. Limitations and Evidence Gaps

Despite increasing interest in IF as a potential metabolic approach for brain health, several significant limitations must be recognised. First, much of the mechanistic evidence linking IF to neuroprotective pathways comes from preclinical studies or short-term human metabolic research [73]. While these studies offer valuable insights into pathways such as mitochondrial regulation, autophagy, and inflammatory modulation, their applicability to long-term neurodegenerative outcomes in humans remains uncertain. Furthermore, human studies on IF are highly diverse in terms of fasting protocols, duration, caloric restriction levels, and participant characteristics, which complicates cross-study comparisons. A significant evidence gap exists concerning the relationship between IF interventions and established fluid biomarkers of neurodegeneration [60]. Although biomarkers such as Aβ42/Aβ40, phosphorylated tau species, NfL, and GFAP are increasingly utilised to monitor early neurodegenerative processes, few controlled human studies have examined whether IF directly influences these markers. Current evidence is therefore predominantly indirect, relying on mechanistic inference or on studies conducted in other neurological conditions. Future research should focus on well-designed longitudinal trials that integrate standardised IF protocols with repeated biomarker measurements, metabolic profiling, and clinical outcomes in populations at risk of NDs [45,62].

## 11. Conclusions

The converging evidence from molecular, metabolic, and clinical studies suggests that IF may represent a biologically based neurometabolic intervention capable of affecting early processes involved in NDs. IF activates evolutionarily conserved pathways that regulate mitochondrial function, autophagy, neuroinflammation, and synaptic plasticity through key mediators like BHB, BDNF, and SCFAs, reflecting coordinated adaptations within the gut–brain axis as observed in preclinical and mechanistic studies [71]. However, much of this evidence derives from experimental models, and its direct translation to human NDs remains to be fully established. These effects go beyond caloric restriction and align with stress-resilience mechanisms that promote neuronal health and influence glial activity. Meanwhile, progress in biomarker research has greatly enhanced the detection of neurodegenerative changes during the prolonged preclinical and prodromal stages. Plasma biomarkers indicating neuronal, glial, and synaptic health, including GFAP, NfL, phosphorylated tau species, SNAP-25, and neurogranin, offer sensitive, clinically accessible tools for early biological assessment, especially when used in combined multimarker panels. Taken together, these findings suggest that integrating IF into a clinical biochemistry framework may help support biomarker-informed research strategies aimed at understanding the metabolic modulation of early neurodegenerative processes. Combining metabolic interventions with sensitive, minimally invasive biomarker assessment supported by ultrasensitive analytical technologies may enable future studies to evaluate personalised metabolic responses to IF while maintaining a proactive focus on preserving metabolic balance and neuronal integrity before the onset of irreversible cognitive decline.

## Figures and Tables

**Table 1:** Principal CSF and plasma biomarkers relevant to early neurodegeneration, highlighting their pathological domains, diagnostic applications, and supporting evidence from recent studies. Abbreviations: AD (Alzheimer’s disease); Aβ42 (Amyloid-β 42); Aβ42/Aβ40 ratio (Amyloid-β 42/amyloid-β 40 ratio); AT(N) (Amyloid–Tau–Neurodegeneration framework); BDNF (Brain-Derived Neurotrophic Factor); CSF (cerebrospinal fluid); GFAP (Glial Fibrillary Acidic Protein); GAP-43 (Growth-Associated Protein 43); hFABP (Heart-type Fatty Acid-binding Protein); IL-6 (Interleukin-6); MCP-1 (Monocyte chemoattractant protein-1); NfL (Neurofilament Light chain); p-Tau181/217/231 (Phosphorylated Tau at threonine 181, 217, and 231); sTREM2 (Soluble Triggering Receptor expressed on myeloid cells 2); SNAP-25 (Synaptosomal-associated Protein 25); S100B (S100 calcium-binding protein B); TDP-43 (TAR DNA-binding protein 43); t-Tau (Total Tau); VILIP-1 (Visinin-like protein 1); YKL-40 (chitinase-3-like protein 1).

Pathological Domain	Biomarker(s)	Biofluid(s)	Main Pathological Process Captured	Clinical/Research Use in Early Disease
Amyloid pathology	Aβ42 Aβ42/Aβ40 ratio	CSF, plasma	Amyloid-β production/clearance imbalance	Identification of amyloidosis; inclusion in multimarker panels for early AD diagnosis
Tau pathology	p-Tau181 p-Tau217 p-Tau231	CSF, plasma	Tau phosphorylation, tangle formation, neuronal injury	Discrimination of AD vs. controls and non-AD dementias; staging along AT(N) framework
	t-Tau	CSF	Neurodegeneration	Marker of neuronal injury and disease severity
Neurodegeneration	NfL hFABP	CSF, plasma	Axonal injury and neurodegeneration	Sensitive markers of neuronal damage; prognosis and disease monitoring across AD and non-AD dementias
	VILIP-1	CSF	Neuronal calcium-mediated injury	Marker associated with neuronal damage and disease progression
Astroglial activation	GFAP	CSF, plasma	Astrocyte reactivity and astrogliosis	Early marker of amyloid-driven astroglial response; prediction of progression in preclinical and prodromal AD
Synaptic dysfunction	SNAP-25 Neurogranin Progranulin GAP-43	CSF	Synaptic loss and dysfunction	Early indication of synaptic damage; improved discrimination between controls and preclinical AD when combined with tau markers
	BDNF	CSF, plasma	Neurotrophic signalling and synaptic plasticity	Exploratory marker of synaptic plasticity and neurotrophic support; currently less established than SNAP-25 and neurogranin
Neuroinflammation	YKL-40 sTREM2 S100B	CSF	Microglial and astroglial activation, inflammatory signalling	Association with imaging measures of neurodegeneration; under evaluation for routine diagnostic use
	IL-6 MCP-1	CSF, plasma	Cytokine-mediated inflammatory signalling	Investigational markers of neuroinflammatory activation
Proteinopathy (non-AT(N) axis)	TDP-43	CSF (experimental)	TDP-43 protein aggregation	Emerging biomarker of TDP-43 proteinopathy; CSF detection remains experimental and assays are not yet standardised

**Table 2:** Illustrative summary of selected metabolic, molecular, and clinical effects of intermittent fasting (IF) reported in representative experimental and clinical studies. This table is not intended to be comprehensive. Abbreviations: IF (intermittent fasting); TRF (time-restricted feeding); ADF (alternate-day fasting); IGF-1 (Insulin-like Growth Factor 1).

Reference	Intermittent Fasting Protocol	Main Outcomes	Molecular/Biological Mechanisms
Brocchi et al., 2022 [33]	Various IF regimens (TRF, ADF)	Improved insulin sensitivity, lipid metabolism and body composition	Improved insulin signalling and mitochondrial efficiency, associated with brain energy metabolism and neuroprotection
Brandhorst et al., 2015 [34]	Periodic fasting-mimicking cycles	Reduced IGF-1 levels, improved metabolic markers	Enhanced cellular stress resistance, regeneration and improved cognitive performance
Joaquim et al., 2022 [35]	IF strategies	Improved glycaemic control and metabolic flexibility	Reduced inflammatory markers and improved metabolic regulation
Silva et al., 2023 [36]	Different IF regimens	Body weight reduction and improved metabolic homeostasis	Regulation of metabolic pathways associated with glucose metabolism and insulin signalling
Punyatoya et al., 2025 [77]	Various IF regimens	Improved glycaemic control, reduced HbA1c, weight loss and improved insulin sensitivity	Enhanced insulin signalling, reduced oxidative stress, circadian rhythm regulation and improved metabolic flexibility

## Data Availability

No new data were created or analyzed in this study. Data sharing is not applicable to this article.

## Abbreviations

The following abbreviations are used in this manuscript:

AD	Alzheimer’s disease
ADF	Alternate-Day Fasting
AI	Artificial Intelligence
Aβ	Amyloid-beta
Aβ42/Aβ40	Amyloid-beta 42/amyloid-beta 40 ratio
AMPK	AMP-activated protein kinase
AT(N)	Amyloid/tau/neurodegeneration framework
BDNF	Brain-derived Neurotrophic Factor
BHB	β-hydroxybutyrate
CSF	Cerebrospinal Fluid
DALYs	Disability-adjusted life years
GABA	Gamma-aminobutyric acid
GAP-43	Growth-associated protein 43
GFAP	Glial Fibrillary Acidic Protein
GH	Growth hormone
hFABP	Heart-type fatty acid-binding protein
IF	Intermittent Fasting
IGF-1	Insulin-like Growth Factor 1
IL-6	Interleukin-6
MAPK	Mitogen-activated protein kinase
MCP-1	Monocyte chemoattractant protein-1
MCI	Mild Cognitive Impairment
mTOR	Mechanistic Target of Rapamycin
NDs	Neurodegenerative diseases
NF-κB	Nuclear factor kappa-light-chain-enhancer of activated B cells
NfL	Neurofilament light chain
PD	Parkinson’s disease
PI3K/Akt	Phosphoinositide 3-kinase/protein kinase B
p-Tau	Phosphorylated tau
p-Tau181	Phosphorylated tau at threonine 181
p-Tau217	Phosphorylated tau at threonine 217
p-Tau231	Phosphorylated tau at threonine 231
RCTs	Randomised controlled trials
ROS	Reactive oxygen species
SCFAs	Short-Chain Fatty Acids
S100B	S100 calcium-binding protein B
SIRT1	Sirtuin 1
SIRT3	Sirtuin 3
SNAP-25	Synaptosomal-Associated Protein 25
sTREM2	Soluble triggering receptor expressed on myeloid cells 2
TDP-43	TAR DNA-binding protein 43
t-Tau	Total tau
TREM2	Triggering receptor expressed on myeloid cells 2
TRF	Time-restricted Feeding
UCH-L1	Ubiquitin C-terminal hydrolase L1
VRF	Visceral fat ratio
VILIP-1	Visinin-like protein 1
YKL-40	Chitinase-3-like protein 1
